# The Prevalence and Determinants of Inappropriate Oral Anticoagulant Use in Patients with Atrial Fibrillation, in Resource-Limited Setting

**DOI:** 10.1155/2023/6673397

**Published:** 2023-12-08

**Authors:** Abdella Birhan Yabeyu, Samiya Yassin Mohammed, Eshetu Shiferaw Legesse, Meaza Adugna, Zemene Demelash Kifle

**Affiliations:** ^1^Department of Pharmacy, College of Medicine and Health Sciences, Ambo University, Ambo, Ethiopia; ^2^Yekatit 12 Hospital Medical College, College of Health Sciences, Clinical Pharmacist, Addis Ababa, Ethiopia; ^3^Department of Pharmacology, School of Pharmacy, College of Medicine and Health Science, University of Gondar, Gondar, Ethiopia

## Abstract

**Introduction:**

Anticoagulation treatment is routinely underused in patients with atrial fibrillation (AF), particularly in settings with limited resources. The current study is aimed at evaluating the appropriateness of oral anticoagulation treatment among AF patients at the Yekatit 12 Hospital Medical College (Y12HMC), Addis Ababa, Ethiopia.

**Methods:**

Institutional-based retrospective cross-sectional study conducted in Y12HMC from November 2019 to March 2020. During the study period, 256 patients' medical records were found; 231 of them met the eligibility criteria and were included in the study. The data were analyzed using SPSS version 25, descriptive statistics were used to summarize the data, and binary logistic regression was performed to identify predictors of inappropriate anticoagulation management.

**Results:**

The majority of study participants were males (55.8%), and over half of them (57.6%) had a nonvalvular type of AF. The majority of patients (61.9%) were receiving anticoagulant treatment, and of them, warfarin was prescribed to most of the study subjects (71.3%). Nearly half (47.6%) of the study participants had inappropriate anticoagulation treatment; among these, the majority of them (69.1%) were from a valvular type of AF. Patients with a valvular type of AF and having the diagnosis of congestive heart disease as comorbidity showed a statistically significant association towards inappropriate anticoagulation management.

**Conclusion:**

According to the study, a significant portion of AF patients received inappropriate anticoagulant treatment, which may play a significant role for the increased risk of stroke in these groups of patients. All of the inappropriate cases were due to failure to start anticoagulant treatment.

## 1. Introduction

Atrial fibrillation (AF) is the most common heart disease associated with significant morbidity and mortality due to stroke and thromboembolism. The annual stroke risk is estimated to be 5–7% in people with AF, although it varies greatly depending on factors such as age, sex, and other medical conditions [1–3]. On the other hand, the use of oral anticoagulants effectively reduces the risk of stroke [2, 4], and numerous studies have demonstrated that anticoagulant medication is effective for long-term stroke prevention [5–9].

Anticoagulant treatment is routinely underutilized in AF patients, with documented percentages nearing 65% [8–11]. Warfarin is the most commonly used anticoagulant in clinical practice, particularly in resource-limited countries such as Ethiopia. It is used in the treatment and prevention of arterial and deep vein thrombosis in individuals with AF [12, 13]. Clinicians' lack of awareness of current recommendations and incorrect risk stratification criteria, or a poor understanding of the risk-benefit ratio, exaggerating the risk of bleeding, could be contributing to the limited use of anticoagulants in AF patients [14, 15].

A patient's annual risk of stroke is estimated using the CHA2DS2-VASc score. The American College of Cardiology (ACC) and the American Heart Association (AHA) recommend using this score for assessing stroke risk in patients with AF [13, 16, 17]. If there are no compelling contraindications, all patients with nonvalvular AF who score ≥ 1 (males) and ≥2 (females) points on the risk score should be treated with anticoagulant therapy to mitigate the risk of stroke. However, patients with cardiomyopathy and valvular AF are advised to initiate anticoagulation treatment regardless of their CHA2DS2-VASc score [18].

Many trials have demonstrated that anticoagulation treatment reduces the estimated yearly risk of stroke in people with AF by two-thirds, and this benefit is accompanied by a relatively low bleeding risk [16, 19]. Despite this, recent data show that the use of anticoagulants in clinical settings is very limited. Consequently, the current study was conducted at the Yekatit 12 Hospital Medical College (Y12HMC) in Addis Ababa, Ethiopia, with the aim of identifying the prevalence and predictors of inappropriate oral anticoagulation management practices among AF patients.

## 2. Methods

### 2.1. Study Design, Study Setting, and Study Period

An institutional-based retrospective cross-sectional study was conducted among AF patients at Y12HMC from November 2019 to March 2020 in Addis Ababa, Ethiopia. Y12HMC is administered by the Addis Ababa Health Bureau. The hospital serves as a catchment area affiliated with Addis Ababa University and provides services to approximately four million people annually. It consists of nine departments and has over 260 beds. The hospital has separate internal medicine wards for male and female patients, and AF patients are admitted to the respective medical wards [20].

### 2.2. Source and Study Population

A patient who presented at Y12HMC with a verified diagnosis of AF was considered part of the source population, whereas AF patients who met the eligibility criteria were included in the study population.

### 2.3. Sample Size Determination

During the study period, medical records of 256 patients were found, with 231 of them meeting the criteria and being included in the study.

### 2.4. Eligibility Criteria

Patients with a confirmed diagnosis of AF based on electrocardiographic findings, who were admitted to Y12HMC between January 2017 and January 2020, were included in this study. This includes patients who were admitted for AF as well as those with an existing AF diagnosis but were admitted for other reasons. Patients with incomplete medical records, on the other hand, were excluded from the study.

### 2.5. Data Collection Instrument and Technique

The data were retrieved from the patients' medical records, and the appropriateness of anticoagulation treatment was evaluated based on ACC and AHA guidelines after a careful assessment of the medical charts. According to the guidelines, patients with nonvalvular AF are not recommended for anticoagulant treatment if they have a CHA2DS2-VASc score of 0 for males or 1 for females. Conversely, individuals with a CHA2DS2-VASc score of ≥1 for males or ≥2 for females should be considered for anticoagulation therapy. Additionally, the guidelines strongly recommend anticoagulation treatment for patients with valvular AF and those with AF and cardiomyopathy, irrespective of their CHA2DS2-VASc scores. The data collection instrument was developed after reviewing several similar articles [9–12, 20–23]. The study instrument is divided into eight separate sections: parts 1 and 2 focus on the sociodemographic and clinical characteristics of study participants, while parts 3, 4, and 5 address the profiles of study participants in terms of comorbidity, types of anticoagulants used (if available), and the list of other drugs used in combination with anticoagulants, respectively. Sections 6 and 7 cover baseline and current laboratory findings, such as coagulation parameters and organ function tests. Finally, part 8 deals with the evaluation of the appropriateness of anticoagulation therapy using ACC and AHA guidelines for patients with AF. Data was collected by two well-trained pharmacists (B.Pharm) and two nurses (BSc). Data collectors underwent a two-day training session to ensure consistency in their understanding and interpretation of the study instrument, as well as uniform implementation of the screening processes of patients' medical records and adherence to data confidentiality issues. Furthermore, the instrument was pretested, and all necessary adjustments and revisions were made prior to the main study's implementation.

### 2.6. Data Analysis

The collected data were analyzed using the Statistical Package for Social Sciences (SPSS, version 25). The data were summarized using descriptive statistics, including mean, standard deviation, percentage, and ranges. Binary logistic regression was used to predict variables associated with the inappropriateness of anticoagulation treatment, with a statistically significant association defined as a *p* value of ≤0.05.

### 2.7. Ethical Consideration

The study was approved by the ethics review committee of Ambo University. Furthermore, the need for ethical consent was waived by the Y12HMC Ethical Review Board, and the current study adheres to the principles outlined in the Declaration of Helsinki. Only numerical identifiers were used for referencing purposes, and the privacy and confidentiality of the subjects were protected by not recording specific identification details such as patients' names and addresses.

### 2.8. Operational Definition

#### 2.8.1. Valvular Atrial Fibrillation

AF patients presented with moderate to severe mitral stenosis or prosthetic heart valves, confirmed via echo/imaging.

#### 2.8.2. A HAS-BLED Score

AF patients were classified as HAS-BLED scores of 0, 1-2, and 3 as having a low, intermediate, or high risk of bleeding, respectively [21].

#### 2.8.3. Appropriate Anticoagulation Treatment

For stroke prevention, patients with nonvalvular AF who have a CHA2DS2-VASc score of 0 for males or 1 for females do not require anticoagulant treatment. However, individuals with a CHA2DS2-VASc score of ≥1 for males or ≥2 for females should be considered for anticoagulation treatment [17]. Anticoagulation treatment is recommended for patients with valvular AF and AF patients with cardiomyopathy, regardless of their CHA2DS2-VASc score [13, 22].

#### 2.8.4. Inappropriate Anticoagulation Treatment

When AF patients are not treated in accordance with the above recommendations, their anticoagulation therapy is considered inappropriate.

## 3. Results

### 3.1. Sociodemographic and Clinical Characteristics

The study included 231 patients who fulfilled the eligibility criteria. The mean age of study participants was 47.2 ± 19.6 years, with a range of 15 to 86. Males constituted the majority of study participants (55.8%), and nearly two-thirds (62.3%) resided in urban areas. Additionally, almost half of the participants (48.9%) reported no history of substance misuse. Furthermore, the nonvalvular type of AF was present in over half of the patients (57.6%) in the study. The most common comorbid conditions among study participants were congestive heart failure (58.0%), chronic valvular heart disease (51.5%), and hypertension (22.7%) (Table 1).

### 3.2. Risk of Stroke and Bleeding

**Figure 1:**
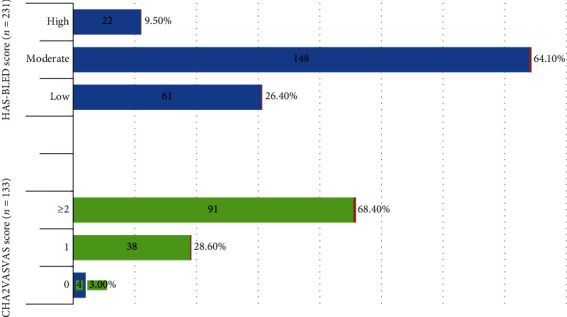
The risk of stroke and bleeding of study participants with AF at the Yekatit 12 Hospital Medical College from November 2019 to March 2020, Addis Ababa, Ethiopia.

The CHA2DS2-VASc and HAS-BLED scores were used to estimate the risk of stroke and bleeding, respectively. The mean CHA2DS2-VASc score for the study participants was 3.8 ± 1.4, with a range of 0 to 7. Over two-thirds (68.4%) of patients with nonvalvular AF had a CHA2DS2-VASc score of ≥2. The mean HAS-BLED score among study participants was 1.6 ± 0.7, ranging from 0 to 5. Almost two-thirds of AF patients (64.1%) were classified as having an intermediate risk of bleeding (Figure 1).

### 3.3. Distribution of International Normalized Ratio (INR) Values

**Figure 2:**
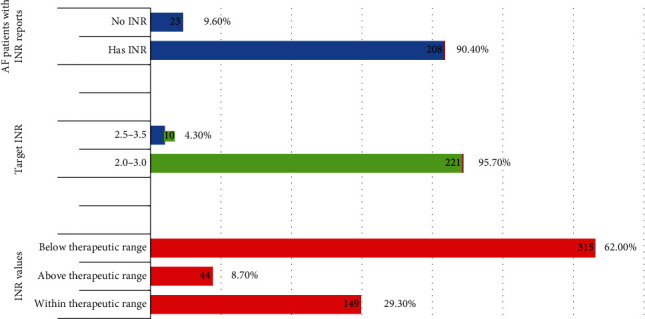
INR records among AF patients at the Yekatit 12 Hospital Medical College from November 2019 to March 2020, Addis Ababa, Ethiopia.

The Rosendaal method was used to calculate the time in the therapeutic range for patients receiving warfarin, with an average duration of 2572.7 ± 764 days. The majority of the AF patients in the study (95.7%) had an INR of 2-3. However, a significant number of patients (9.6%) were unable to afford routine INR follow-up. Among the study subjects, 508 INR tests were performed, with a mean ± SD INR value of 8.4 ± 2.3, ranging from 1.1 to 9.7. About 29.3% of these results were within the therapeutic range, while the remaining INR values were either below or above the therapeutic range (Figure 2).

### 3.4. Types of Medications Prescribed along with Anticoagulants

Along with anticoagulants, a variety of medications were prescribed. Furosemide, enalapril, and spironolactone were the most commonly used medications among study participants, accounting for 71.9%, 66.7%, and 44.2%, respectively (Table 2).

### 3.5. Method and Types of Anticoagulation

The majority of AF patients (61.9%) were receiving anticoagulant treatment. Among them, warfarin was prescribed to 71.3% of the study participants, and 6.3% received both warfarin and aspirin. During the review of the patients' medical records, it was found that 25 of them had an absolute contraindication to oral anticoagulant medication (Table 3).

### 3.6. Evaluation of Appropriateness of Anticoagulation Treatment

The study revealed that 47.6% of the study participants had inappropriate anticoagulation treatment. Among these, the majority (69.1%) had the valvular type of AF. All inappropriate cases were due to the failure to start anticoagulant treatment. Patients with apparent contraindications to anticoagulant treatment, such as recent major surgery, severe thrombocytopenia, and severe anemia, who had not initiated anticoagulants at the time of hospital discharge, were considered to have appropriate treatment (Table 4).

### 3.7. Predictive Factors towards Inappropriate Anticoagulation Management Practice

Binary logistic regression was performed to identify determinants of inappropriate anticoagulation management practices in AF patients. In multivariate logistic regression, the type of AF and the comorbidity of congestive heart failure showed a statistically significant association with inappropriate anticoagulant practice. Patients with valvular AF had a 52.2% greater risk of inappropriate anticoagulation treatment compared to patients with nonvalvular AF (AOR = 0.488, CI: 0.651-0.982, *p* value = 0.004). AF patients without congestive heart failure had an 85.4% lower risk of inappropriate anticoagulation treatment compared to AF patients with this comorbidity (AOR = 0.146, CI: 0.803-7.541, *p* value = 0.002) (Table 5).

## 4. Discussion

The aim of the study was to determine the appropriateness of anticoagulation management practices among AF patients at Y12HMC in Addis Ababa, Ethiopia. The ACC and AHA guidelines were utilized to determine the appropriateness of anticoagulation treatment. According to the current study, 47.6% of AF patients had inappropriate anticoagulant treatment. Various studies conducted worldwide have reported a prevalence of inappropriate anticoagulation treatment in AF patients ranging from 34% to 64% [8–11, 24–29].

In comparison to the current study, a higher rate of inappropriate anticoagulation management practices was reported. In a study conducted at the Gondar University Hospital in Ethiopia, approximately two-thirds (64.78%) of AF patients were receiving inappropriate anticoagulant treatment [28]. Likewise, in a Korean study, oral anticoagulants were underutilized in 64% of patients [8]. The difference in study designs and the varied degree of risk levels among study participants may have contributed to the increased magnitude of inappropriate anticoagulation management practices among AF patients in the above studies.

In this study, approximately one-third (30.9%) of patients with nonvalvular AF received inappropriate anticoagulant treatment. A similar finding was reported from the University of Wisconsin Hospital and Clinics in Madison, Wisconsin. In that study, 134 patients with nonvalvular AF were included, with 34% of the participants receiving inappropriate anticoagulant therapy [27].

The INR range should be between 2.0 to 3.0 in most circumstances, although there are a few exceptions. For instance, in cases where warfarin is administered for prevention after a myocardial infarction or in patients with mechanical prosthetic heart valves, the range should be 2.5 to 3.5 [30–32]. As noted in their medical records, a significant number of AF patients (9.6%) in the study were unable to undergo INR monitoring tests due to financial restrictions. As a result, clinicians were reluctant to administer anticoagulant treatment for these groups of patients due to concerns about bleeding. Similarly, another study reported that 9.0% of AF patients could not afford regular INR tests [29].

The binary regression results demonstrated that patients with valvular AF and those with congestive heart failure as a comorbidity had a positive association with inappropriate anticoagulant treatment among AF patients, which is consistent with findings from other studies [9, 33]. This finding emphasizes the significance of healthcare providers exercising increased vigilance in monitoring patients with valvular AF and congestive heart failure. Regular follow-ups and medication adjustments are essential to ensure appropriate anticoagulation therapy. Additionally, further research is required to comprehend the underlying reasons for this connection and enhance treatment strategies and patient outcomes in the future. Previous studies have linked inappropriate anticoagulant treatment to a history of stroke or transient ischemic attack, the risk of bleeding, advanced age (>80 years), and being female [8, 10, 26, 28]; however, none of the above variables played a part in this study.

The study was a single-centered study, so caution needs to be taken while extrapolating the study findings, and the total number of study participants was small compared to some other studies. However, based on the existing evidence, this study provided credible information on the appropriateness of anticoagulant management practices in AF patients.

## 5. Conclusion

The study revealed that a large number of AF patients received inappropriate anticoagulant treatment, which may significantly contribute to the increased risk of stroke for this group of patients. Patients with valvular AF and those with a comorbidity of congestive heart disease showed a significant association with inappropriate anticoagulant treatment.

## Figures and Tables

**Table 1:** Demographic and clinical characteristics of AF patients at the Yekatit 12 Hospital Medical College from November 2019 to March 2020, Addis Ababa, Ethiopia.

Demographic and clinical characteristics	Frequency (%)
*Age*	
15-29	63 (27.3)
30-59	122 (52.8)
>60	46 (19.9)

*Gender*	
Male	129 (55.8)
Female	102 (44.2)

*Residence*	
Rural	87 (37.7)
Urban	144 (62.3)

*Substance abuse*	
No substance abuse	113 (48.9)
Daily smokers	34 (14.7)

*Alcohol (n* = 84)	
Occasional drinkers	51 (22.1)
Most days of the week	33 (14.3)

*Types of AF*	
Nonvalvular	133 (57.6)
Valvular	98 (42.4)

*Comorbid disease*	
Chronic rheumatoid valvular heart disease	119 (51.5)
Uncontrolled hypertension SBP (≥160)	64 (22.7)
Diabetes mellitus	29 (12.6)
Chronic kidney disease	15 (6.5)
Stroke	49 (21.2)
Congestive heart failure	134 (58.0)
Chronic kidney disease	4 (1.7)
Hyperthyroidism	38 (16.5)
Degenerative valvular heart disease	25 (10.8)
Cardiomyopathy	2 (0.87)
S.cr.≥2.26	7 (3.0)
AST/ALT/AP > 3x normal	2 (0.9)
^∗^Others	12 (5.2)

SBP = systolic blood pressure, S.cr = serum creatinine, AST = aspartate aminotransferase, and ALP/AP = alkaline phosphate. ^∗^Included acute coronary syndrome, asthma, and peripheral arterial disease.

**Table 2:** Medications prescribed together with anticoagulants among AF patients at the Yekatit 12 Hospital Medical College from November 2019 to March 2020, Addis Ababa, Ethiopia.

Medications	Frequency (%)
Amlodipine	84 (36.4)
Atenolol	42 (18.2)
Atorvastatin	39 (16.9)
Benzathine penicillin	67 (29.0)
Carvedilol	9 (3.9)
Digoxin	14 (6.1)
Enalapril	154 (66.7)
Furosemide	166 (71.9)
Hydrochlorothiazide	61 (26.4)
Insulin	29 (12.6)
Metformin	37 (16.0)
Metoprolol	82 (35.5)
Spironolactone	102 (44.2)
Others^∗^	24 (10.4)

^∗^Propylthiouracil, omeprazole, and propranolol.

**Table 3:** Type and method of anticoagulation among AF patients at the Yekatit 12 Hospital Medical College from November 2019 to March 2020, Addis Ababa, Ethiopia.

Type and methods of anticoagulation	Frequency	%
No anticoagulation	88	38.1
Anticoagulation	146	61.9
Aspirin	32	21.9
Warfarin	102	69.8
Rivaroxaban	3	2.1
Both aspirin and warfarin	9	6.2
Reasons for failure to initiate anticoagulants (*n* = 25)		
Severe thrombocytopenia (<50 platelets/lL)	14	63.6
Severe anemia (<7.0 hemoglobin g/dl.)	8	36.4
Recent surgery	3	12.0

**Table 4:** Evaluation of anticoagulation management practice among AF patients at the Yekatit 12 Hospital Medical College from November 2019 to March 2020, Addis Ababa, Ethiopia.

Anticoagulation management practice	Frequency	%
Appropriate	121	52.4
Inappropriate	110	47.6
Inappropriate from valvular AF	76	69.1
Inappropriate from nonvalvular AF	34	30.9

**Table 5:** Binary logistic regression for predictive factors towards inappropriate anticoagulation management practice among AF patients at the Yekatit 12 Hospital Medical College from November 2019 to March 2020, Addis Ababa, Ethiopia.

Variable	Appropriate vs inappropriate anticoagulation, *n* (%)		Crude OR (CI 95%)	Adjusted OR (CI 95%)	*p* value
Types of AF					
Valvular	22	76	**1.00**		
Nonvalvular	99	34	0.352 (0.247-0.519)	0.488 (0.651-0.982)^∗^	**0.004 ** ^∗^
CHA2DS2-VASc score					
1	9	1	**1.00**		
2	44	13	1.948 (0.620-1.185)	1.753 (0.418-2.238)	
≥3	46	20	8.473 (0.199-10.32)	1.638 (0.399-6.719)	
Chronic valvular heart disease					
Yes	107	6	**1.00**		
No	124	9	8.188 (0.968-7.407)	9.109 (0.503-6.866)	
Hypertension					
Yes	64	5	**1.00**		
No	167	7	8.386 (3.143-8.292)	10.36 (0.346-2.639)	
Diabetes mellitus					
Yes	29	2	**1.00**		
No	202	3	7.651 (0.635-5.809)	3.363 (0.754-6.547)	
Chronic kidney disease					
Yes	15	1	**1.00**		
No	216	2	3.139 (0.754-5.505)	4.299 (0.565-3.103)	
Stroke					
Yes	6	2	**1.00**		
No	225	4	0.175 (0.374-4.221)	1.95 (0.794-6.529)	
Congestive heart disease					
Yes	82	24	**1.00**		
No	149	35	0.36 (4.795-11.47)	0.146 (4.803-7.541)^∗^	**0.002 ** ^∗^
Hyperthyroidism					
Yes	38	1	**1.00**		
No	193	4	8.473 (0.969-6.282)	1.638 (0.339-7.371)	
Degenerative valvular heart disease					
Yes	25	2	**1.00**		
No	206	3	1.01 (0.704-4.340)	0.961 (0.545-2.078)	
Type and method of anticoagulation					
No anticoagulant	0	88	**1.00**		
Warfarin	102	0	0.316 (0.293-2.812)	0.939 (0.933-6.584)	
Aspirin	10	22	0.85 (0.682-1.804)	0.614 (0.551-1.902)	
Both warfarin and aspirin	9	0	0.266 (0.923-1.911)	0.57 (0.263-3.142)	

^∗^Statistically significant association with inappropriate anticoagulation management practice.

## Data Availability

The data used to support the findings of this study are available from the corresponding author on reasonable request.

## Abbreviations

ACC: American College of Cardiology
AF: Atrial fibrillation
AHA: American Heart Association
INR: International normalized ratio
Y12HMC: Yekatit 12 Hospital Medical College.
